# Management of thyroid emergencies: joint consensus statement on management of thyroid storm*

**DOI:** 10.1530/ETJ-26-0043

**Published:** 2026-08-19

**Authors:** Peter Taylor, Onyebuchi Okosieme, Grigoris Effraimidis, Fraser Gibb, Dhruv Parekh, Carla Moran, Peter Kopp, Kristien Boelaert

**Affiliations:** ^1^ Department of Immunology and Immunotherapy, School of Infection, Inflammation and Immunology, College of Medicine and Health, University of Birmingham, Birmingham, UK; ^2^ Department of Endocrinology, University Hospital of Wales, Cardiff, UK; ^3^ Thyroid Research Group, Systems Immunity Research Institute Cardiff University School of Medicine, Cardiff, United Kingdom; ^4^ Department of Endocrinology and Metabolic Diseases, Larissa University Hospital, Faculty of Medicine, School of Health Sciences, University of Thessaly, Larissa, Greece; ^5^ Edinburgh Centre for Endocrinology & Diabetes, Royal Infirmary of Edinburgh, Edinburgh, UK; ^6^ Birmingham Acute Care Research Group, Institute of Inflammation and Ageing, University of Birmingham, Birmingham, UK; ^7^ School of Medicine, University College Dublin, Dublin, Ireland; ^8^ Division of Endocrinology, Diabetology and Metabolism, University of Lausanne, Lausanne, Switzerland; ^9^ Department of Applied Health Research, School of Health Sciences, College of Medicine and Health, University of Birmingham, Birmingham, UK

**Keywords:** thyroid emergencies, guidelines, thyroid storm, thyrotoxicosis, Burch/Wartofsky, Society for Endocrinology, British Thyroid Association, European Thyroid Association

## Abstract

Thyroid storm is a rare but life-threatening endocrine emergency characterised by extreme thyrotoxicosis and multi-organ dysfunction. Prompt recognition and rapid, coordinated management are essential to reduce its high mortality. This consensus statement, developed by experts in endocrinology and acute care, provides practical recommendations for the diagnosis and treatment of thyroid storm. Diagnosis remains clinical, aided by validated scoring systems such as the Burch–Wartofsky and Japanese Thyroid Association/Japan Endocrine Society criteria, which also predict outcomes. Management requires a multidisciplinary approach in high-dependency or intensive care settings, incorporating supportive measures, aggressive treatment of precipitating factors, and targeted therapies. Core treatments include beta-blockade, high-dose antithyroid drugs, corticosteroids, and iodide, with careful attention to timing and contraindications. Adjunctive therapies, such as cholestyramine, lithium, plasmapheresis, and extracorporeal membrane oxygenation (ECMO), as well as surgical thyroidectomy in selected cases, may be required for refractory disease. This guidance also highlights the need for definitive management of underlying hyperthyroidism after stabilisation. By summarising current evidence and expert consensus, this document aims to standardise care, support clinical decision-making, and improve outcomes in patients presenting with thyroid storm.

## Introduction

Thyroid storm is an acute, life-threatening, hypermetabolic state, caused by severe manifestations of thyrotoxicosis. It requires prompt recognition and aggressive management to mitigate its high mortality rate (1). Fortunately, it is rare, and a precise estimate of its true incidence is difficult due to considerable variability in the criteria for its diagnosis, which is based on clinical rather than biochemical grounds (2). However, thyroid storm represents a clinically significant proportion of hospitalisations among individuals admitted with severe thyrotoxicosis (10–16%) (1). There is no serum thyroid hormone level or specific test that delineates severe thyrotoxicosis from thyroid storm.

Graves’ disease is the most frequently reported underlying aetiology of thyroid storm in most case series and registry studies, although toxic multinodular goitre and, less commonly, destructive thyroiditis have also been described (2, 3). Patient-related risk factors include older age, long-standing or poorly controlled hyperthyroidism, and non-adherence to antithyroid therapy. Precipitating events are common and include infection, surgery, trauma, myocardial infarction, stroke, parturition, diabetic ketoacidosis, and exposure to iodine-containing contrast agents (2). Cardinal features suggestive of thyroid storm include fever (usually above 38.5°C), tachycardia that is disproportionate to the level of fever, arrhythmia (particularly atrial fibrillation), cardiac decompensation and pulmonary oedema, altered mental status, and gastrointestinal dysfunction (3, 4, 5). Given that progression from severe thyrotoxicosis to thyroid storm may occur rapidly in the presence of such stressors, early recognition of clinical deterioration and prompt initiation of aggressive therapy are critical to prevent multi-organ dysfunction and reduce mortality.

The pathophysiology of thyroid storm is incompletely understood. Serum thyroid hormone concentrations are often similar to those observed in patients with severe but uncomplicated thyrotoxicosis, and there is considerable biochemical overlap between groups (2, 6). The proposed mechanisms include heightened tissue responsiveness to catecholamines, activation of inflammatory cytokine pathways, relative predominance of triiodothyronine (T3)-mediated effects at the tissue level, and possible alterations in thyroid hormone binding and cellular uptake (4). These factors likely interact to produce a hypermetabolic state with multi-organ dysfunction.

Reported mortality rates in thyroid storm vary depending on case definition and illness severity, ranging from approximately 8% to over 30% in published series. In a multicentre French intensive care unit (ICU) cohort of 92 patients with definite thyroid storm requiring intensive care admission, in-ICU mortality was 17%, with 6-month mortality of 22% (7). Cardiogenic shock within the first 48 h of ICU admission was independently associated with in-ICU mortality (odds ratio: 9.43), as was greater non-cardiovascular organ dysfunction as reflected by Sequential Organ Failure Assessment score. These findings underscore the prognostic importance of early cardiac dysfunction and multi-organ failure. Other studies have similarly identified advanced age, need for mechanical ventilation, renal replacement therapy, and pre-existing cardiac disease as adverse prognostic factors. While most survivors recover without permanent sequelae, prolonged ICU admission and severe hypoperfusion may result in persistent neurological impairment in a minority of cases. Early recognition, aggressive supportive care, and prompt control of thyrotoxicosis are therefore central to improving survival. Disseminated intravascular coagulation is also increasingly recognised as a serious complication of thyroid storm and should be considered in patients who develop unexplained bleeding, bruising, or coagulopathy (14). In addition, socioeconomic deprivation and lack of health insurance have been independently associated with markedly higher rates of hospitalisation for thyrotoxicosis and thyroid storm; clinicians should be alert to the possibility of under-recognition and delayed presentation in under-served populations (14).

This consensus statement covers the diagnostic scoring criteria for thyroid storm and the key principles of management, namely general supportive measures, beta-blockade, antithyroid drugs, glucocorticoid therapy, and iodine.

## Methods

This document represents an expert consensus clinical guidance statement developed following nomination of panel members by the European Thyroid Association, British Thyroid Association, Society for Endocrinology, and Welsh Endocrine and Diabetes Society. Panel members were selected based on recognised expertise in thyroid disease, endocrine emergencies, and acute/critical care management.

A structured literature review was undertaken using PubMed/MEDLINE from January 1990 to June 2025 with search terms including ‘thyroid storm’, ‘thyrotoxic crisis’, ‘antithyroid drugs’, ‘beta-blocker’, ‘iodine’, ‘plasmapheresis’, ‘thyroidectomy’, and ‘ECMO’. Existing international guidelines were reviewed in detail. Draft recommendations were prepared and iteratively revised until unanimous agreement was achieved. Given the rarity of thyroid storm and predominance of observational data, formal evidence grading (e.g. GRADE) was not undertaken. Recommendations therefore reflect integration of available evidence with expert clinical consensus.

### Recognition and diagnosis

Thyroid storm is a clinical diagnosis. The scoring systems – Burch and Wartofsky (6) (Table 1) and a scale proposed by the Japanese Thyroid Association and Japan Endocrine Society (JTA/JES) (8) (Table 2) – are helpful adjuncts in diagnosing thyroid storm (9). These scores are also predictive of mortality and length of stay in intensive care and in hospital (3).

**Table 1:** Burch and Wartofsky scoring system (6).

Criteria	Score
Temperature	
37.2–37.7°C	5 points
37.8–38.2°C	10 points
38.3–38.8°C	15 points
38.9–39.4°C	20 points
39.5–39.9°C	25 points
≥40.0°C	30 points
Central nervous system disturbance	
Absent	0 points
Mild (agitation)	10 points
Moderate (delirium, psychosis, extreme lethargy)	20 points
Severe (seizure, coma)	30 points
Gastrointestinal–hepatic dysfunction	
Absent	0 points
Moderate (diarrhoea, abdominal pain, nausea/vomiting)	10 points
Severe (jaundice)	20 points
Cardiovascular dysfunction	
Tachycardia (beats/min)	
<90	0 points
90–109	5 points
110–119	10 points
120–129	15 points
130–139	20 points
≥140	25 points
Congestive heart failure	
Absent	0 points
Mild (pedal oedema)	5 points
Moderate (bibasilar rales)	10 points
Severe (pulmonary oedema)	15 points
Atrial fibrillation	
Absent	0 points
Present	10 points
Precipitating event*	
Absent	0 points
Present	10 points
Total	
Unlikely to represent thyrotoxic crisis	<25 points
Suggestive of impending crisis	25–44 points
Highly suggestive of thyrotoxic crisis^†^	≥45 points

* A precipitating event in patients with untreated or inappropriately treated hyperthyroidism: acute infection, trauma, surgery, childbirth, ketoacidosis, myocardial infarction, stroke or transient ischaemic attack, radioiodine treatment (rarely), or administration of iodinated contrast media.

^†^ Rapid, aggressive, specific, and supportive treatment required.

**Table 2:** Japanese Thyroid Association/Japan Endocrine Society Thyroid Storm Score (8).

Thyrotoxicosis (as evidenced by elevated serum FT3 and/or FT4) is a prerequisite, and it requires various combinations of the following symptoms:
• CNS manifestation (restlessness, delirium, psychosis/mental aberration, lethargy/somnolence, coma) • Fever (38°C/100.4 F or greater) • Tachycardia (130/min or higher) • CHF (pulmonary oedema, rales, cardiogenic shock, or NYHA class IV) • GI/hepatic manifestation (nausea, vomiting, diarrhoea, total bilirubin 3 mg/dL or more)
Diagnosis
Definite thyroid storm (TS1)
Thyrotoxicosis (elevated FT3 and/or FT4) plus
• At least one CNS manifestation, plus one or more other symptoms (fever, tachycardia, CHF, GI/hepatic) ‘OR’ a combination of at least three features among fever, GI/hepatic, CHF, or tachycardia
Suspected thyroid storm (TS2)
Thyrotoxicosis (elevated FT3 and/or FT4) plus
• A combination of at least two features among tachycardia, CHF, GI/hepatic, and fever ‘OR’ a patient with h/o thyroid disease, presence of goitre, and exophthalmos who meets criteria for TS1 but TFTs not available

A Burch and Wartofsky score of <25 points suggests thyroid storm is unlikely, a score of 25–44 points is suggestive of a possible impending crisis, and a score of ≥45 is highly suggestive of a thyroid crisis. A decision to instigate the aggressive treatment utilised in managing thyroid storm in a patient with a score of 25–44 should be undertaken by an experienced clinician/intensivist in conjunction with an endocrinologist. The JTA/JES score (8) provides a diagnosis of definite thyroid storm (TS1) or suspected thyroid storm (TS2 (Table 3)).

**Table 3:** Comparison of diagnostic criteria for thyroid storm.

Feature	BWPS	JTA/JES criteria
Basis	Expert opinion-derived numerical score (1993)	Nationwide registry-derived criteria (2012; revised 2016)
Biochemical thyrotoxicosis required	No	Yes
Structure	Weighted point scale across organ systems	Categorical combination of defined clinical features
Diagnostic threshold	≥45 highly suggestive; 25–44 supportive	‘Definite’ vs ‘suspected’ based on feature combinations
Strength	Flexible; may prioritise sensitivity	Structured; may prioritise specificity
Limitation	Subjective weighting; fever and tachycardia may reduce specificity in ICU/sepsis; overlap with severe thyrotoxicosis	Categorical rigidity; may under-recognise early or evolving cases
Role in practice	Adjunct to clinical judgement	Adjunct to clinical judgement

BWPS, Burch–Wartofsky Point Scale.

While both the Burch–Wartofsky Point Scale (BWPS) and the JTA/JES criteria are widely used for diagnosing thyroid storm, neither has been validated against a gold standard diagnostic test. The BWPS assigns weighted scores to thermoregulatory, cardiovascular, gastrointestinal, and central nervous system manifestations. However, its reliance on clinical parameters, such as fever and tachycardia, may lead to overestimation of thyroid storm in critically ill patients with alternative causes of systemic inflammatory response, particularly in ICU settings. The heavy weighting of hyperthermia and heart rate has been criticised as potentially lacking specificity in septic or cardiogenic shock.

The JTA/JES criteria require biochemical confirmation of thyrotoxicosis and defined combinations of organ system manifestations. These criteria were derived from nationwide registry data and have demonstrated associations with mortality in observational cohorts. However, their categorical structure may under-recognise (6) evolving or incomplete presentations.

In ICU populations in particular, caution is warranted to avoid overdiagnosis in patients with non-thyroidal critical illness who have coincidental biochemical hyperthyroidism.

Published data on sensitivity and specificity of these systems are limited, and both tools exhibit overlap with severe but uncomplicated thyrotoxicosis. As such, diagnostic scoring systems should be regarded as structured clinical aids rather than definitive diagnostic instruments. Clinical judgement, trajectory of deterioration, and response to therapy remain central to diagnosis.

Clinicians should also be aware that older patients with thyroid storm may present atypically, with a paucity of the classic adrenergic features of thyrotoxicosis (the so-called apathetic hyperthyroidism”), including blunted tachycardia, minimal agitation, and vague or non-specific symptoms. A high index of suspicion is therefore required in this population, and thyroid function testing should be considered even when the clinical picture is not classic (10).

Some key differential diagnoses to consider when thyroid storm is suspected are shown in Box 1.

Box 1 **Key differential diagnoses of presenting symptoms of thyroid storm.**

• **Severe or uncontrolled thyrotoxicosis**

– Marked biochemical hyperthyroidism without significant central nervous system dysfunction or multi-organ failure

• **Sepsis**

– Fever, tachycardia, and systemic inflammatory response with an identifiable infectious focus; elevated inflammatory markers

• **Encephalitis/meningitis**

– Altered consciousness with meningeal signs, focal neurological findings, or cerebrospinal fluid abnormalities

• **Hypertensive encephalopathy**

– Severe hypertension with neurological symptoms (headache, confusion, visual disturbance) and absence of biochemical thyrotoxicosis

• **Heatstroke**

– Hyperthermia following environmental exposure or exertion, often with anhidrosis and history of heat exposure

• **Sympathomimetic drug toxicity (e.g. cocaine, amphetamines)**

– Severe agitation, hypertension, and tachycardia with relevant drug exposure history

• **Neuroleptic malignant syndrome**

– Recent exposure to dopamine antagonists, generalised ‘lead-pipe’ rigidity, and elevated creatine kinase

### Treatment

**Figure 1:**
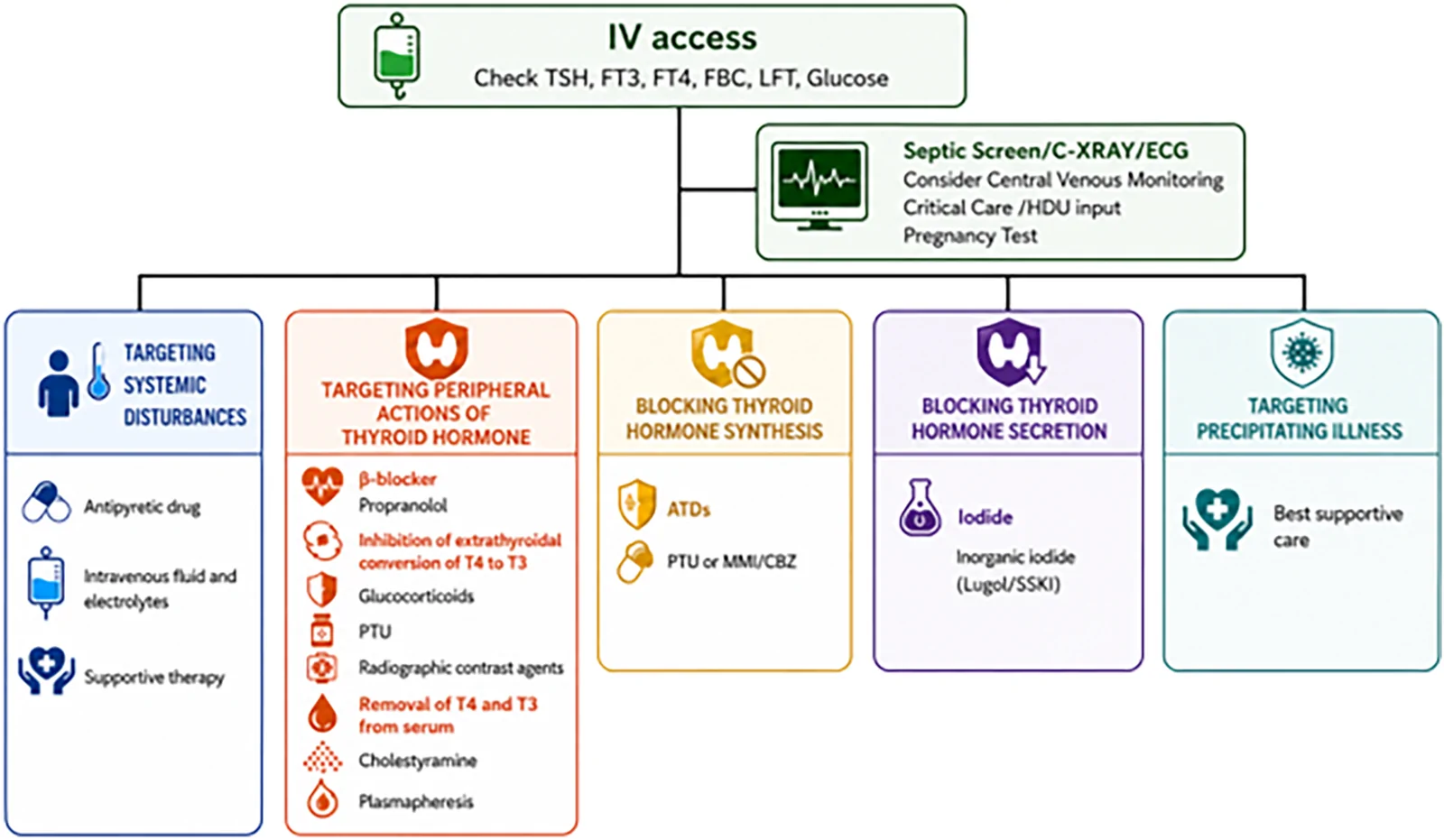
Overview of treatment strategies for thyroid storm. ATD, antithyroid drug, PTU, propylthiouracil; MMI, methimazole; CBZ, carbimazole; SSKI, saturated solution of potassium iodide; C-XRAY, chest X-ray; ECG, electrocardiogram, HDU, high-dependency unit.

A broad overview of treatment is shown in Fig. 1 based on the American Thyroid Association (ATA) guidelines (5). Similar recommendations are available from the European Thyroid Association (ETA) (11). Treatment is targeted at correcting systemic disturbances, reducing peripheral action of thyroid hormones, reducing thyroid hormone synthesis and secretion, and treatment of the precipitating event. Once stabilised, patients are maintained on antithyroid drugs and usually should be advised to have definitive treatment for hyperthyroidism (radioactive iodine therapy or surgery).

Patients with suspected or confirmed thyroid storm should be managed in a high-dependency or intensive care setting when there is evidence of significant organ dysfunction or clinical instability. Suggested criteria for ICU admission include the following:

- Haemodynamic instability requiring vasopressor support or invasive monitoring.
- Altered level of consciousness, stupor, or coma.
- Respiratory compromise requiring non-invasive or invasive ventilatory support.
- Acute heart failure, cardiogenic shock, or persistent clinically significant arrhythmia.
- Evidence of multi-organ dysfunction.
- Rapid clinical deterioration despite initial therapy.

### General measures

- A multidisciplinary team approach is important to successfully offer all necessary therapeutic options.
- Patients should be managed in a high-dependency unit (HDU) or intensive care unit (ICU) setting for intensive monitoring and treatment.
- Need to seek ICU expertise early, and also ensure surgeons are made aware of the patient so they can assess and prepare for emergency thyroidectomy if this is required.
- Initial management includes appropriate supportive care, including cooling, paracetamol, and appropriate intravenous (IV) fluids.
- Treat any precipitant aggressively (e.g. antibiotics for infection).
- A pregnancy test should be performed in all women of childbearing potential as part of the initial workup (Fig. 1). Thyroid storm in pregnancy is rare but carries substantial maternal and fetal risk, and management should involve early liaison with obstetric and neonatal teams (10).
- Atrial fibrillation is common in thyroid storm; anticoagulation decisions should follow standard stroke-risk stratification (e.g. CHA2DS2-VASc), balanced against bleeding risk, noting that many patients revert to sinus rhythm once euthyroidism is restored (10, 12).
- Be vigilant that these patients are at increased risk of requiring respiratory support.
- Chlorpromazine 50–100 mg IM can be considered if sedation is required; this will also reduce temperature.
- Close monitoring of glucose levels is required – patients are at risk of hypoglycaemia.
- Nutritional support, including vitamin replacement (e.g. thiamine), must be considered. Aspirin should be avoided as it can increase circulating thyroid hormone levels. It competes with thyroid hormones for binding to plasma proteins, including thyroxine-binding globulin, resulting in transient increases in circulating free T4 and free T3 concentrations. Paracetamol (acetaminophen) is therefore preferred for antipyresis.

### Beta-blockade

- Propranolol 60–80 mg every 4 h is recommended in the ATA and ETA guidelines (5, 11). These high doses also have a potential advantage in reducing T4-to-T3 conversion.
- Intravenous rather than oral route is an option if the patient is unstable or unable to take medication orally due to vomiting/agitation (Box 2).
- Consider invasive monitoring in patients with a history of or suspected congestive heart failure.
- Consider cautious lower initial doses of beta-blockers as beta-blockade can potentially precipitate cardiovascular collapse, especially in patients with systolic heart failure.
- Consider IV esmolol instead of propranolol as this has the advantage of a short half-life and can be rapidly discontinued if adverse outcomes occur (5, 8). Many clinicians use esmolol rather than propranolol for this reason.
- Diltiazem may be of benefit if beta-blockers are contraindicated (e.g. asthma, cardiac decompensation under beta-blocker therapy); it can be given 60–90 mg orally every 6 h and can also be given IV.

However, beta-adrenergic blockade remains a cornerstone of therapy in thyroid storm, reducing adrenergic activity and, with propranolol, peripheral T4-to-T3 conversion. In haemodynamically stable patients without significant left ventricular dysfunction, non-selective beta-blockers (e.g. propranolol) are effective for rate control. However, in patients with suspected thyrotoxic cardiomyopathy, hypotension, or evolving cardiogenic shock, caution is required, as beta-blockade may precipitate haemodynamic collapse by removing compensatory adrenergic support. In such cases, a short-acting agent such as intravenous esmolol is preferred due to its titratability. Early assessment of cardiac function, including echocardiography, is recommended to guide therapy.

Some retrospective data have suggested increased mortality with propranolol compared with beta1-selective agents (e.g. landiolol, bisoprolol) in patients with heart failure, which underpins the JTA/JES preference for beta1-selective agents. However, a subsequent larger cohort study found comparable in-hospital mortality between propranolol and beta1-selective beta-blockers, including in patients with acute heart failure. Agent selection should therefore be individualised according to local availability and patient factors rather than a single preferred drug (12).

Box 2 **Possible IV beta-blocker regimes.**

**Propranolol:** 1–3 mg max rate of 1 mg/min IV. Repeat only once after 2 min. Infusion can be started at 2–3 mg/hr.

**Labetalol:** 5–20 mg IV over 2 min; then 0.5 mg/min.

**Esmolol:** Loading dose: 500 μg/kg over 1 min (70 kg = 35 mg) IV. Maintenance is 0–200 μg/kg/minute (70 kg = 0–14 mg/min).

### Antithyroid drugs

High-dose antithyroid (ATD) drug therapy is a central component of thyroid storm management, aiming to rapidly inhibit new thyroid hormone synthesis and, in the case of propylthiouracil (PTU), also reduce peripheral conversion of T4 to T3. High doses are recommended based on the need to achieve prompt and near-complete inhibition of thyroid peroxidase activity in the setting of life-threatening decompensation. Recent observational data comparing PTU and MMI in critically ill patients with thyroid storm did not demonstrate a clear mortality advantage of PTU over MMI (13). Although PTU has the theoretical advantage of inhibiting peripheral T4-to-T3 conversion, this has not translated into clear superiority in outcome studies.

Liver enzyme abnormalities are common in severe thyrotoxicosis and thyroid storm and may reflect hepatic congestion, hypoxia, or direct thyrotoxic effects. Mild-to-moderate transaminase elevation does not automatically contraindicate ATD therapy. However, given the recognised risk of severe hepatotoxicity with PTU, clinically significant hepatic dysfunction – for example, transaminases exceeding approximately three times the upper limit of normal or rising bilirubin – should prompt strong consideration of MMI/CBZ in preference to PTU, unless specific circumstances (e.g. first trimester pregnancy) dictate otherwise (5). In cases of acute liver failure or severe hepatic dysfunction, specialist hepatology input is recommended.

We therefore propose the following:

- MMI 60–80 mg/day or CBZ 80–100 mg/day as these appear to have an equivalent efficacy in thyroid storm compared with PTU (13). In particular, strongly consider MMI/CBZ rather than PTU if disturbed liver function is indicated (3x the upper limit of normal for bilirubin/liver enzymes). In some European countries, IV MMI is available (Thiamazol 40 mg/mL). OR Propylthiouracil (PTU) 500–1,000 mg loading dose oral/NG/PR and then 250 mg every 4 h.
- The potential advantage of PTU over methimazole (MMI)/carbimazole (CBZ) is the additional benefit of peripheral blockade of T4-to-T3 conversion.
- Lower the dose of antithyroid drugs as soon as practically possible to PTU 400 mg a day/MMI 30 mg a day/CBZ 40 mg a day.
- Cholestyramine 4 g three times a day may be useful; it increases clearance of thyroid hormones by blocking enterohepatic circulation, although this is an off-label use.
- Lithium can be considered, but toxicity may be an issue; specialist advice should be sought before considering this. A starting lithium dose of 300 mg BD, which can be increased, is reasonable.

### Corticosteroids

- IV hydrocortisone 100 mg loading dose and then 50 mg four times a day.
- High-dose steroid therapy decreases conversion of T4 to T3.
- These may be particularly effective if Graves’ disease is the underlying cause.
- High-dose glucocorticoids, as used in thyroid storm, have been reported to precipitate thyrotoxic periodic paralysis in poorly controlled thyrotoxic patients. Serum potassium should be monitored closely after glucocorticoid administration, particularly in at-risk groups (e.g. young Asian or Hispanic men) (12).

### Iodine/potassium iodide

Iodine therapy is most effective in hyperthyroidism driven by increased thyroid hormone synthesis, such as Graves’ disease or toxic multinodular goitre, where it induces the acute Wolff–Chaikoff effect and inhibits further hormone release.

In destructive thyroiditis (e.g. subacute or painless thyroiditis, amiodarone-induced type 2 thyrotoxicosis) and in factitious thyrotoxicosis, excess circulating hormone results from the release of preformed hormone or exogenous intake rather than ongoing synthesis; in these settings, iodine therapy is unlikely to be beneficial. In amiodarone-induced type 1 thyrotoxicosis, which reflects iodine-induced increased hormone synthesis, the additional benefit of iodide administration may be limited due to the already high iodine load, and its use should be considered cautiously alongside high-dose antithyroid drugs.

We therefore propose the following if iodine is to be used:

- Wait for 1 h post-administration of antithyroid drugs before administering iodide – this delay is essential as thyrotoxicosis could otherwise worsen depending on the aetiology.
- Give as Lugol’s iodine (iodine/potassium iodide solution) 8 drops four times a day or as a saturated solution of potassium iodide (SSKI) 5 drops four times a day. Discontinue once the patient is stabilised.
- Iodine solutions can be locally irritant and must be diluted (for example in milk or orange juice) before oral/NG administration.
- Be aware that, depending on the aetiology of the thyrotoxicosis, the Wolff–Chaikoff effect is transient (escape phenomenon).
- Iodine administration can suppress thyroidal uptake through saturation of intrathyroidal iodine stores, and it may interfere with the effectiveness of subsequent radioactive iodine therapy. To allow washout of excess iodine and recovery of radioiodine uptake, radioiodine is generally deferred for at least 2–3 months.

### Ongoing management

- A marked improvement in thyroid storm usually occurs within 24–72 h. Once haemodynamic, thermoregulatory, and neurological stability has been achieved, attention should switch to maintenance therapy.
- The most relevant indicators include resolution of hyperthermia, reduction in heart rate, improvement in haemodynamic stability (reduced vasopressor requirement if applicable), recovery of mental status, and improvement in gastrointestinal dysfunction.
- A progressive decline in free T3 and free T4 concentrations is expected over several days. Free T3 may fall more rapidly where PTU or corticosteroids are used. TSH should not be used to guide early treatment response.
- Failure to demonstrate clinical stabilisation within 24–48 h should prompt reassessment for ongoing precipitating factors, inadequate drug delivery (e.g. impaired gastrointestinal absorption), cardiomyopathy, or the need for escalation of therapy.
- Iodide therapy should be stopped as soon as possible when hormone levels are under control, but beta-blockade should be used while the patient remains thyrotoxic.
- Corticosteroid therapy may be stopped once the patient is stabilised and if there is no suspicion of accompanying adrenal insufficiency.
- Antithyroid treatment should be continued until a final decision regarding long-term antithyroid drug therapy, surgery, or radioiodine (RAI) therapy can be made.
- Some patients may require thyroidectomy during the admission for thyroid storm. If this has not been undertaken, definitive treatment options for management of thyrotoxicosis should be considered and discussed prior to discharge. Preferred options are thyroidectomy or RAI therapy, although in some cases, long-term antithyroid drugs may be used.

In patients who fail to stabilise despite prompt initiation of standard therapy (antithyroid drugs, beta-blockade where appropriate, corticosteroids, iodine, and supportive care), a structured escalation approach is recommended.

- Optimisation of first-line therapy

Before proceeding to advanced interventions, clinicians should confirm adequate dosing and absorption of antithyroid drugs, ensure appropriate timing of iodine administration, reassess for ongoing precipitating factors, and optimise haemodynamic and ventilatory support. Impaired gastrointestinal absorption in critically ill patients should be considered.

(ii) Adjunctive pharmacologic measures

Cholestyramine may be added to enhance enterohepatic clearance of thyroid hormones. In selected cases, glucocorticoid dosing may be intensified. These measures are adjunctive rather than definitive rescue therapies.

(iii) Established rescue therapies for refractory instability

Therapeutic plasma exchange (plasmapheresis) may be considered in patients with severe, refractory thyrotoxicosis or multi-organ failure despite maximal medical therapy. Although evidence is limited to case series and observational data, plasma exchange can transiently reduce circulating thyroid hormone levels and may serve as a bridge to definitive therapy. Both plasmapheresis/plasma exchange and emergency thyroidectomy have been used to treat thyroid storm (5).

Emergency thyroidectomy should be considered in specialised centres when medical therapy fails or is contraindicated. Surgical intervention is associated with significant perioperative risk and should occur with experienced endocrine surgical and critical care teams. In selected high-risk patients who cannot tolerate general anaesthesia, thyroidectomy can be performed with local anaesthesia and sedation/hypnosis by an experienced endocrine surgeon (14).

Venoarterial extracorporeal membrane oxygenation (VA-ECMO) may be appropriate in cases of refractory cardiogenic shock, particularly where severe thyrotoxic cardiomyopathy is present. ICU cohort data indicate that cardiogenic shock is a major determinant of mortality, and ECMO may provide haemodynamic stabilisation while definitive endocrine control is achieved. ECMO has been successfully used in thyroid storm (15).

### Rarely used or context-specific therapies

Agents such as perchlorate or iodinated radiographic contrast agents (e.g. iopanoic acid) have been reported in isolated cases but are not widely available and lack robust outcome data. Their use should be considered highly context-dependent and typically restricted to specialist centres. Perchlorate and iopanoic acid may have a role in thyroid storm. However, they are not routinely available, and their use should only be considered under the guidance of those experienced in managing thyroid storm. Case reports and case series suggest perchlorate may be considered as an additional therapeutic option in patients requiring intensification of therapy, but only as an add-on in severe cases where it reduces thyroid hormone secretion. Similarly, iopanoic acid is hard to source and is unlicensed; however, some laboratory-grade iopanoic acid, which is fit for human consumption, has been utilised and has proved life-saving in selected cases. Iopanoic acid profoundly inhibits thyroid hormone release, as well as peripheral conversion of thyroxine to triiodothyronine, resulting in a dramatic and rapid fall in thyroid hormone levels.

## Declaration of interest

The authors declare that there is no conflict of interest that could be perceived as prejudicing the impartiality of the work reported.

## Funding

This work did not receive any specific grant from any funding agency in the public, commercial, or not-for-profit sector.

## Disclaimer

The document should be considered as an expert consensus only; it is not intended to determine an absolute standard of medical care. The clinical team concerned must make the management plan fully considering the individual circumstances of each patient.
